# Efficiency and Safety of the Selective Relaxant Binding Agent Adamgammadex Sodium for Reversing Rocuronium-Induced Deep Neuromuscular Block: A Single-Center, Open-Label, Dose-Finding, and Phase IIa Study

**DOI:** 10.3389/fmed.2021.697395

**Published:** 2021-08-25

**Authors:** Yanhua Zhao, Sifan Chen, Xiaorong Huai, Zhangjie Yu, Youmao Qi, Jie Qing, Weifeng Yu, Diansan Su

**Affiliations:** ^1^Department of Anesthesiology, Renji Hospital, Shanghai Jiao Tong University School of Medicine, Shanghai, China; ^2^Hangzhou Adamerck Pharmlabs Inc., Hangzhou, China

**Keywords:** neuromuscular blocking agents, phase IIa study, adamgammadex, selective relaxant binding agents, dose-finding clinical trials

## Abstract

**Background:** Rapid reversal of neuromuscular block after surgery and anesthesia is often necessary. Here, we reported the primary efficacy and safety data from a phase IIa study on adamgammadex sodium, a newly developed modified γ-cyclodextrin derivative.

**Methods:** This was a phase IIa, single-center, randomized, open-label, and dose-finding study that enrolled 35 patients under general anesthesia who received the neuromuscular blocking agent rocuronium for induction and maintenance of neuromuscular blockade. The subjects were randomized to one of the five adamgammadex dose groups (2, 4, 6, 8, and 10 mg kg^−1^) and to the 4 mg kg^−1^ sugammadex group. Pharmacological efficacy was the recovery time from the start of adamgammadex or sugammadex administration to train-of-four (TOF) ratio ≥0.9, 0.8, and 0.7 among the different dose groups. Adverse events were recorded throughout the study.

**Results:** The efficacy in reversing deep neuromuscular block was the same between 4 mg kg^−1^ sugammadex and adamgammadex. However, in the lowest dose groups of 2 and 4 mg kg^−1^ adamgammadex, adequate reversal could not be achieved in all subjects. The recovery time of TOF ratio to 0.9, 0.8, and 0.7 was shorter in the adamgammadex 10 mg kg^−1^ group than in the sugammadex 4 mg kg^−1^ group. The average values of the TOF ratio after 3 min of administration of adamgammadex 8 and 10 mg kg^−1^ and sugammadex 4 mg kg^−1^ were >90%. There were no serious adverse events after the use of adamgammadex, and no subjects had to be withdrawn from the trial.

**Conclusions:** Adamgammadex enabled quick, predictable, and tolerable reversion of rocuronium-induced deep neuromuscular block in a dose-dependent manner. Adamgammadex doses of 6–10 mg kg^−1^ might be the recommended dose range for further exploration of efficacy. **Clinical Trial Registration:** This study was registered at chictr.org.cn, identifier: ChiCTR2000038391.

## Introduction

The routine use of neuromuscular blocking agents (NMBAs) facilitates tracheal intubation and mechanical ventilation, thereby improving the quality of operation, especially abdominal and thoracic surgery (1–3). However, residual neuromuscular blockade (NMB) following surgery and anesthesia may prolong the recovery time and lead to a variety of adverse events, including respiratory complications, hypoxemic episodes, and pharyngeal and upper esophageal dysfunction (4, 5).

The traditional NMBA reversal drugs neostigmine and edrophonium are acetylcholinesterase (AChE) antagonists, which have limited efficacy in reversing profound and deep levels of NMB. In addition, they have effects on the muscarinic acetylcholine receptors and nicotinic receptors in other tissues and may cause relevant side effects, including bradycardia, salivation, bronchospasm, and vomiting (6, 7). In order to limit these side effects, these medications are usually administered in combination with atropine, which may also cause side effects, such as tachycardia, dry mouth, and blurred vision (8).

Sugammadex is the first of the selective relaxant binding agents (SRBAs) to be commercially available after clinical approval in Europe in 2008 and was designed to reverse NMB by forming a complex in a 1:1 ratio with the aminosteriod molecules of NMBAs, such as rocuronium or vecuronium (9–13). Several studies have approved sugammadex to be capable of quickly reversing shallow and deep levels of aminosteroid NMB. However, major concerns about hypersensitivity and anaphylaxis delayed the approval of sugammadex in the United States (14). Approximately 8 years after the initial application for approval to the Food and Drug Administration, sugammadex received approval in 2015. Furthermore, sugammadex increases the potential risk of post-operative bleeding and recurrence of NMB (15, 16).

As a modified γ-cyclodextrin derivative, adamgammadex sodium shares a similar mechanism of action with sugammadex (17, 18). In pre-clinical animal studies, adamgammadex was found to have a concentration-dependent effect of reversing rocuronium-induced NMB in beagle dogs and had significantly low tendency for hypersensitivity and anaphylaxis and negligible detrimental effects on cardiac function and coagulation in zebrafish tests (18). Moreover, results of phase I clinical trials on adamgammadex were encouraging, showed no serious drug-related adverse events in healthy volunteers, and demonstrated stable pharmacokinetics regardless of the dose (19). Notably, adamgammadex and the adamgammadex–rocuronium complex are exclusively excreted through the kidneys.

In this study, we assessed the efficacy and safety of five doses of adamgammadex and the active control drug sugammadex in reversing rocuronium-induced deep NMB of I–II post-tetanic counts (PTCs) in anesthetized patients who underwent elective surgeries. Therefore, our primary objectives were to assess the effects of different doses of adamgammadex (2, 4, 6, 8, and 10 mg kg^−1^) in reversing profound rocuronium-induced NMB and to compare these doses with the well-established use of 4 mg kg^−1^ sugammadex. In addition, we aimed to provide primary efficacy and safety data on adamgammadex and to serve as a pilot study for guiding future development studies.

## Methods

We conducted a single-center, randomized, open-label, active-controlled, parallel group, dose-finding, phase IIa trial at Renji Hospital, Shanghai JiaoTong University, Shanghai, China. The protocol was approved by the Ethics Committee of Renji Hospital (approval number: [2018] 209) and was registered at chictr.org.cn (registration number: ChiCTR2000038391). Subjects were enrolled in our trial between June 2019 and January 2020 after written informed consent was obtained. The inclusion criteria were men or women aged 18–64 years, ASA 1–2, body mass index (BMI) <30 kg m^2^, weight ≥50 kg for men and ≥45 kg for women, and underwent elective surgery under general anesthesia with the use of rocuronium to facilitate tracheal intubation and maintain muscle relaxation. The exclusion criteria were anatomical deformity that was expected to make intubation difficult or allergy to cyclodextrin or other drugs; intake of medications, such as antispasmodics, aminoglycoside antibiotics, and magnesium, which are known to interfere with the study drugs; significant cardiovascular disease, neuromuscular disorder, liver or renal dysfunction, or coagulation disorder; and pregnant or preparing for pregnancy and breastfeeding during the trial. After obtaining informed consent, we carried out comprehensive screening examinations, including demographic data, physical examination, vital signs, BMI, laboratory tests, and 12-lead electrocardiogram (ECG).

The study comprised the core study period 1 and the exploratory study period 2. The initial dose of adamgammadex in the core study period 1 was set to 2, 4, and 6 mg kg^−1^, based on our previous findings in the phase I trial (19). In exploratory study period 2, the investigators reevaluated the efficiency of adamgammadex in different dose groups in reversing profound rocuronium-induced NMB and conducted subsequent dose-finding test or dose escalation test. Twelve subjects were allocated in the higher adamgammadex dose groups (8 and 10 mg kg^−1^): six subjects in the 8 mg kg^−1^ group and another six subjects in the 10 mg kg^−1^ group.

Overall, the subjects were enrolled and allocated to one of the five adamgammadex dose groups (2, 4, 6, 8, and 10 mg kg^−1^) or to the 4 mg kg^−1^ sugammadex active control group by computer randomization. Propofol and sufentanil were used to induce anesthesia and intravenous remifentanil and propofol were used to maintain anesthesia, with dose adjustments of sufentanil to the desired clinical effect. Neuromuscular response was monitored using the TOF-Watch SX acceleromyograph (Organon Ireland Ltd., Ireland). Prior to the administration of rocuronium, stabilization and calibration of the TOF-Watch SX acceleromyograph were accomplished by the attending anesthesiologists. Rocuronium 0.6 mg kg^−1^ was given intravenously, followed immediately by tracheal intubation when muscle relaxation was maximal. Consistent train-of-four (TOF) stimulation was performed every 15 s throughout the surgery; when there was no TOF response detected, the depth of NMB was assessed by 10–15 s PTC stimulation. An extra dose of rocuronium 0.1–0.2 mg kg^−1^ was given if the PTC was >2, with adjustments for surgical need. At the end of the surgical procedure, the subjects were administered a single scheduled intravenous dose of open-label adamgammadex or sugammadex when the TOF-Watch SX reading was 1–2 PTCs. Subjects remained tracheally intubated, sedated, and ventilated to ensure full recovery from the NMB (TOF ratio >0.9 for at least three times for more than 30 min) and to allow complete evaluation of consciousness, respiration, and muscle strength. If the TOF ratio did not reach 0.9 for over 30 min after adamgammadex or sugammadex administration, the subjects were given neostigmine 0.04 mg kg^−1^ and atropine intravenously.

Basic vital signs, including body temperature, blood pressure, heart rate, and respiratory rate, were recorded during the screening period (days – 14–0) and the next day after drug administration. Oxygen saturation was monitored for at least 60 min after recovery to a TOF ratio of 0.9. Blood pressure, pulse oximetry, and heart rate were monitored throughout the surgery; 12-lead ECG was recorded preoperatively, 2–6 h, and the next day after the study drug administration. Laboratory tests, including routine blood tests, coagulation function, urinalysis, and biochemical examination, were measured during the screening period and on follow-up visit. Adverse events that were new in onset or aggravated in severity or frequency within 7 days after the initial dose of the study drug were designated as treatment-emergent adverse events (TEAEs). The intensity of TEAEs was classified according to the common terminology criteria version 4.03. Safety assessments among the groups were concluded depending on the number and proportion of subjects who experienced TEAEs and on the severity and frequency of adverse events.

Recovery from the NMB was studied in the full analysis set (FAS), which comprised all randomized subjects in the intent-to-treat population who received the study drug and were evaluated for change from baseline at least one time, and in the per-protocol set (PPS), which comprised a subset of subjects who entered FAS and had no major protocol violations, good drug compliance, and no use of prohibited drugs and data on the major efficacy variables consistent with the regimen. The safety assessment set (SAS) comprised all patients who received a dose of the study drug.

Primary pharmacological efficacy was evaluated by comparing the recovery time from the start of administration of adamgammadex or sugammadex to TOF ratio ≥0.9 among the different dose groups. Secondary efficacy was the time from the start of administration of adamgammadex to recovery of the TOF ratio to 0.7–0.8. Differences were compared among groups using an analysis of covariance model. Descriptive statistics, including mean, standard deviation, median, quartile, minimum, and maximum values, were grouped by the dose of adamgammadex. Dose–response curves were estimated for the primary and secondary efficacy variables. A two-tailed *p*-value of < 0.05 was considered statistically significant.

Adverse events were concluded according to the System Organ Class and Preferred Term, and intergroup comparison of safety assessments was performed using the Fisher's exact test. Laboratory values, vital signs, and 12-lead ECG readings were summarized descriptively at baseline and after drug administration for each scheduled visit.

## Results

**Figure 1:**
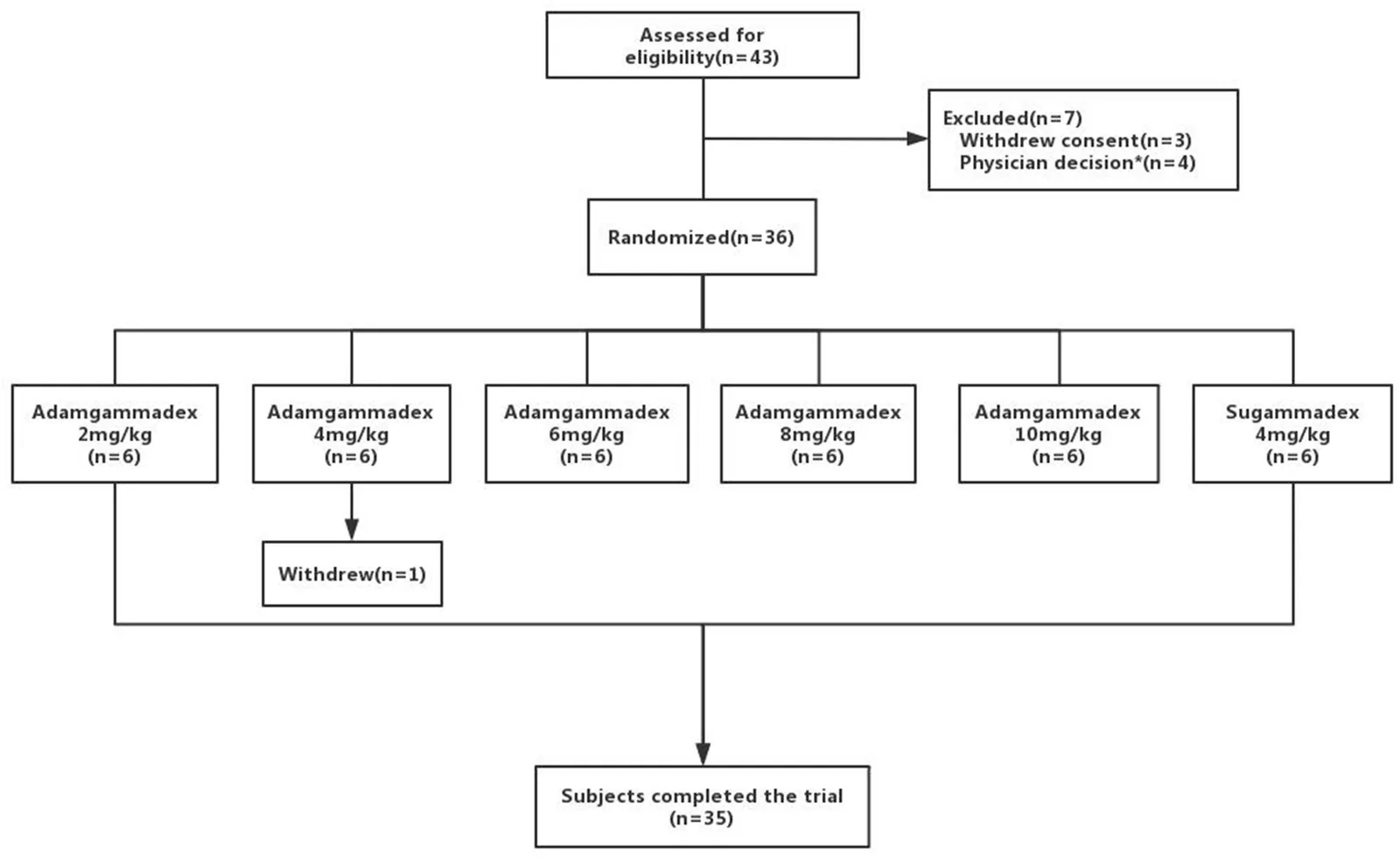
Distribution of subjects. * Including inappropriate management during surgery (*n* = 2) and T1 that was not constantly within the standard deviation range (100% ± 5%) for more than 3 min before rocuronium injection (*n* = 2).

Of the 43 subjects who signed the informed consent, 36 were enrolled and randomized to the study drug groups (Figure 1). Because this was a phase IIa pilot dose-finding study, the sample size was based on practical and empiric considerations, as well as in reference to previous studies, and not based on statistical power calculations. One patient withdrew from the trial before the administration of adamgammadex because of a muscle relaxation monitoring PTC of >2 after surgery, leaving 35 subjects in the SAS. There were 35 subjects enrolled in this study; all dose groups of adamgammadex had 29 cases and the sugammadex 4 mg kg^−1^ group had 6 cases. The demographic and baseline characteristics of the randomized subjects set are shown in Table 1 and were not significantly different among the groups. Three subjects had a history of medication-associated allergy and none had a history of smoking, drug abuse, or alcohol abuse.

**Table 1:** Subjects' demographic characteristics and baseline data.

	**Adamgammadex**					**Sugammadex**
	**2 mg/kg**	**4 mg/kg**	**6 mg/kg**	**8 mg/kg**	**10 mg/kg**	**4 mg/kg**
	**(*n* = 6)**	**(*n* = 6)**	**(*n* = 6)**	**(*n* = 6)**	**(*n* = 6)**	**(*n* = 6)**
Age, y	47.5 ± 13.63	45.0 ± 13.45	40.2 ± 14.47	35.8 ± 11.82	39.5 ± 11.67	52.2 ± 10.40
Sex(male/female), *n*	3/3	1/5	3/3	3/3	4/2	5/1
Height, cm	163.3 ± 8.76	163.2 ± 6.31	168.3 ± 6.38	166.3 ± 9.85	168.2 ± 9.45	166.2 ± 6.52
Weight, kg	64.00 ± 10.00	64.17 ± 11.37	66.50 ± 11.91	67.67 ± 13.49	69.00 ± 17.70	67.75 ± 5.19
BMI, kg/m^2^	23.8 ± 2.32	24.0 ± 2.61	23.5 ± 3.21	24.5 ± 2.81	23.8 ± 3.87	24.8 ± 2.04

*Data are presented as mean ± SD or absolute numbers*.

Table 2 shows the first dose, total additional dose, and total dose of rocuronium in the 35 subjects enrolled in the SAS. Eight subjects had at least one major protocol violation and were excluded from the FAS group (*n* = 27). The major protocol violations comprised the following: failure to antagonize deep muscle relaxation and need for neostigmine for remedy (three subjects in the adamgammadex 2 mg kg^−1^ group) and technical problems with the TOF-Watch SX monitoring groups of adamgammadex 4 mg kg^−1^ (*n* = 1), 6 mg kg^−1^ (*n* = 2), and 8 mg kg^−1^ (*n* = 1) and sugammadex 4 mg kg^−1^ (*n* = 1).

**Table 2:** Dosage of muscle relaxant rocuronium.

	**Adamgammadex**					**Sugammadex**
	**2 mg/kg**	**4 mg/kg**	**6 mg/kg**	**8 mg/kg**	**10 mg/kg**	**4 mg/kg**
	**(*n* = 6)**	**(*n* = 5)**	**(*n* = 6)**	**(*n* = 6)**	**(*n* = 6)**	**(*n* = 6)**
Total dose (mg)	86.2 ± 31.66	104.6 ± 14.47	102.3 ± 29.49	108.5 ± 20.25	92.8 ± 38.91	75.5 ± 15.90
First dose (mg)	38.5 ± 6.22	38.4 ± 7.44	40.0 ± 6.99	40.8 ± 8.18	41.5 ± 10.56	40.8 ± 3.13
total additional dose (mg)	47.7 ± 26.81	66.2 ± 20.18	62.3 ± 30.48	67.7 ± 16.02	51.3 ± 28.81	34.7 ± 14.51

*Data are presented as mean ± SD*.

### Efficacy

**Figure 2:**
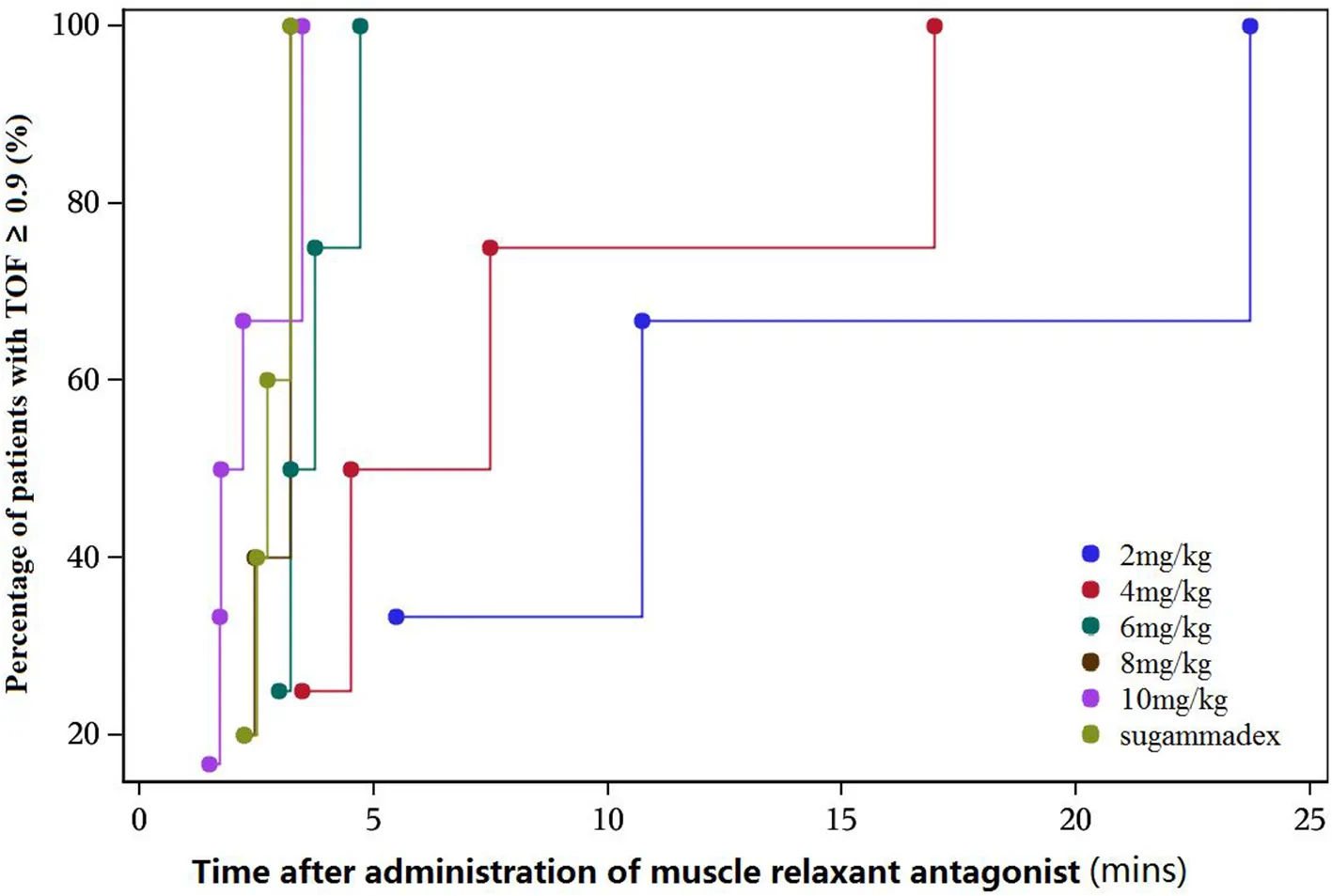
Cumulative percentage of patients recovered [train-of-four (TOF) ratio ≥ 0.9]–recovery time curve.

**Figure 3:**
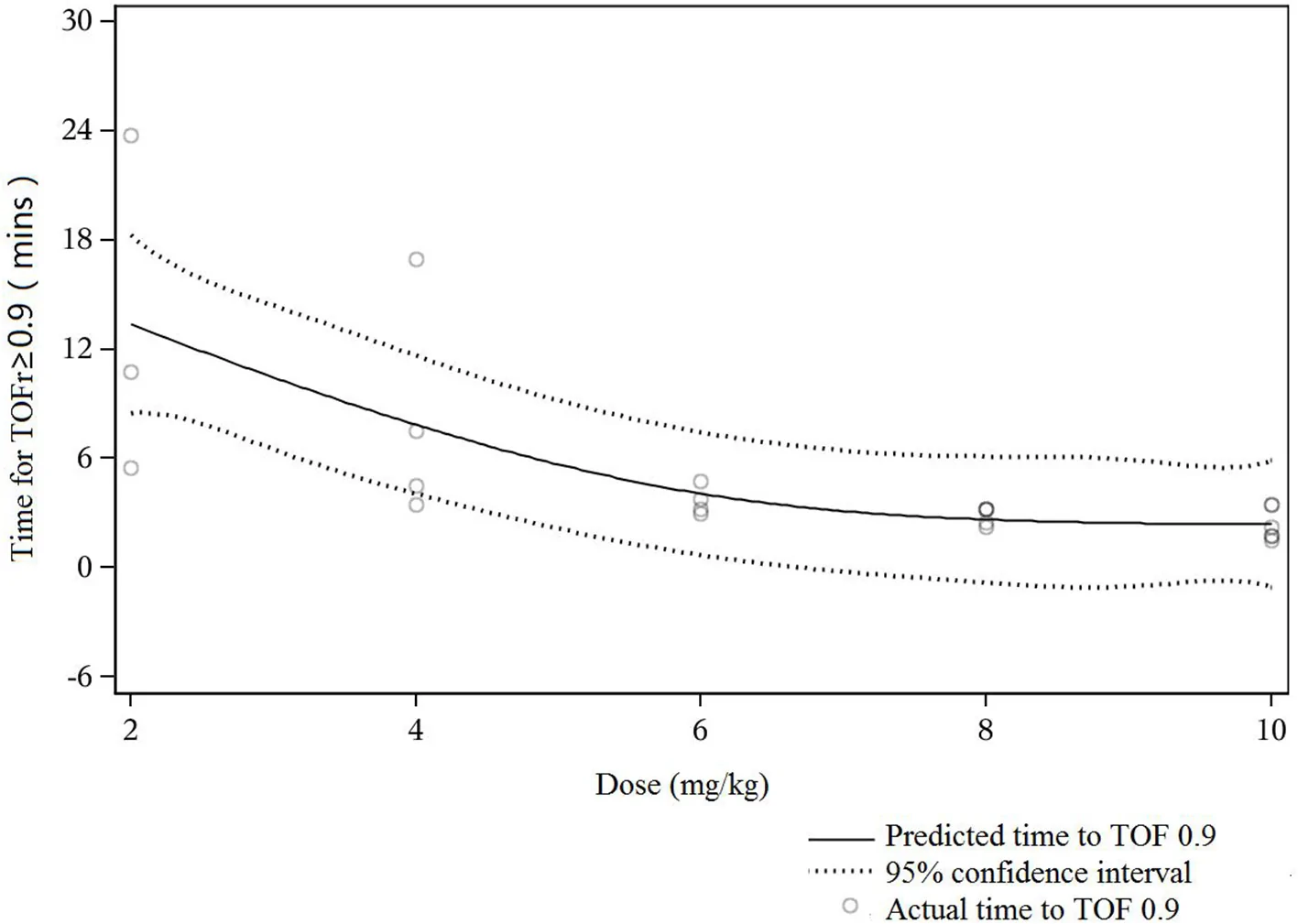
Dose–response curve of muscle relaxant antagonist [train-of-four (TOF) ratio ≥ 0.9].

**Figure 4:**
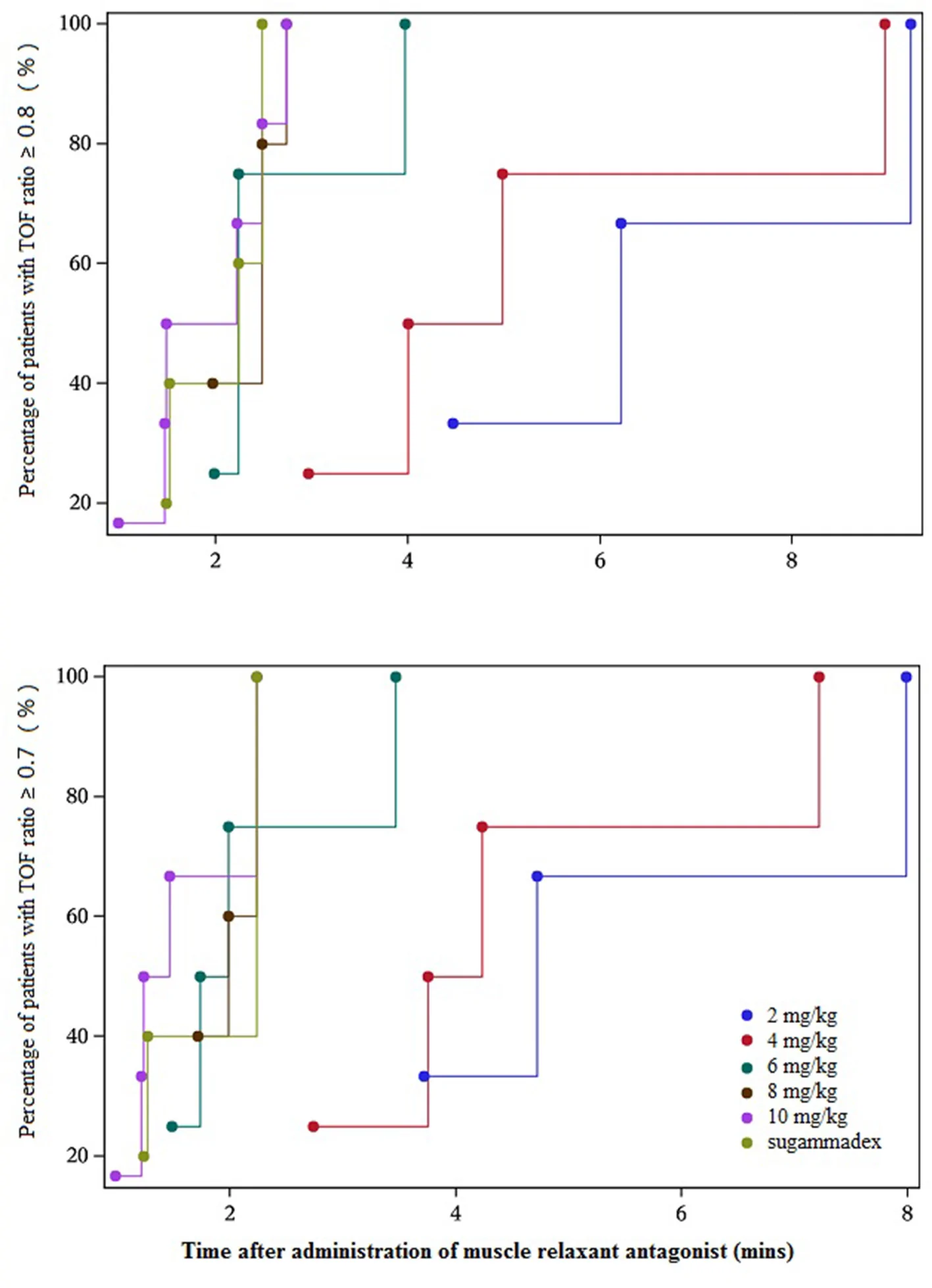
Cumulative percentage of patients recovered [train-of-four (TOF) ratio ≥ 0.8 and 0.7]–recovery time curve.

**Figure 5:**
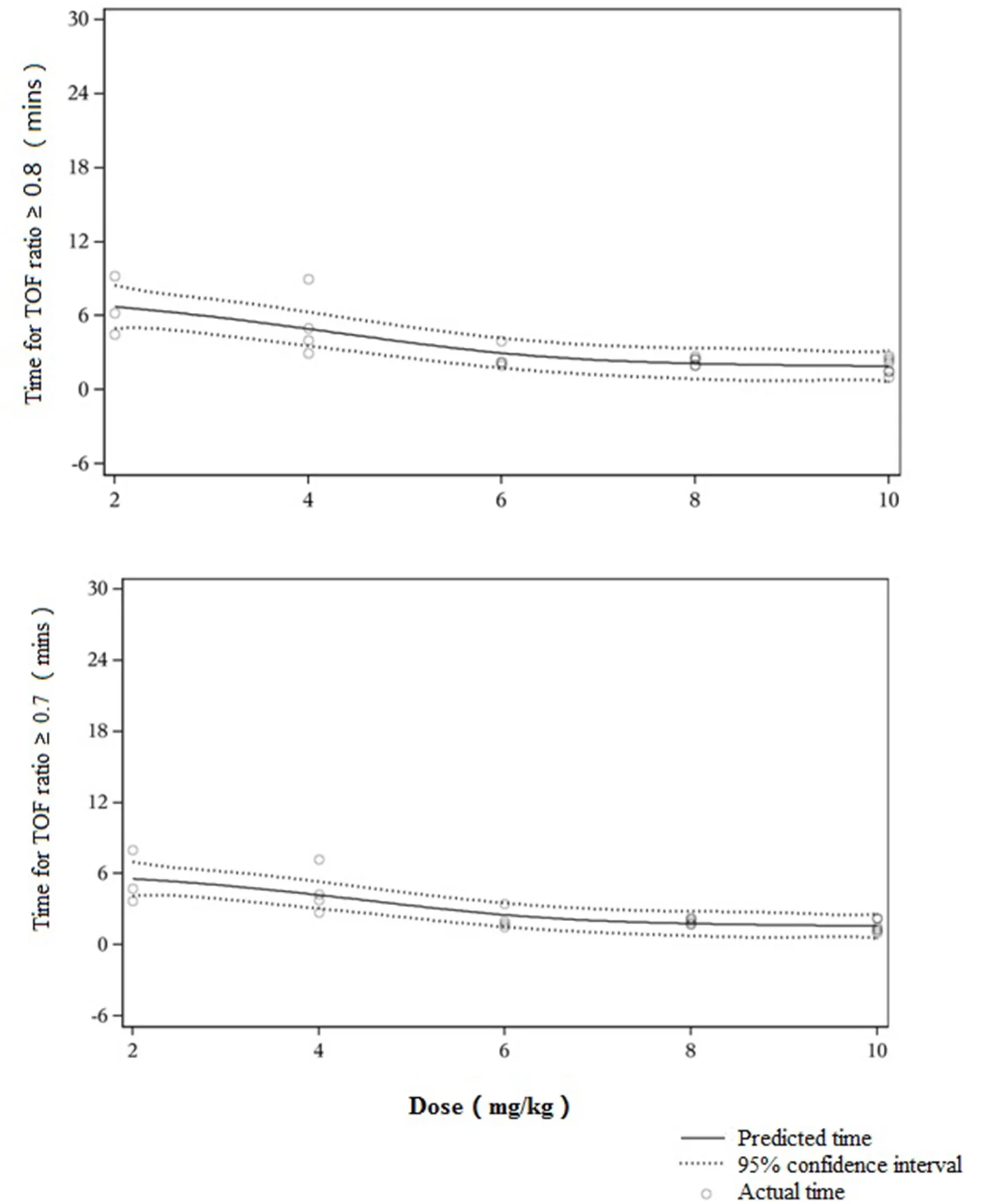
Dose–response curve of muscle relaxant antagonist [train-of-four (TOF) ratio ≥ 0.8 and 0.7].

From the initial single intravenous injection of adamgammadex at 2, 4, 6, 8, or 10 mg kg^−1^ when the PTC was 1–2, the time to recovery of the TOF ratio to 0.9 was dose-dependent, and there was a distinct decrease in the recovery time with increasing doses of adamgammadex (Table 3; Figure 2). Compared with the recovery time of TOF ratio to 0.9 in the active control group of sugammadex 4 mg kg^−1^, those for the adamgammadex 2 and 4 mg kg^−1^ dose groups were significantly prolonged (0.0050 and 0.0271, respectively; *p* < 0.05), and those for the adamgammadex 6 and 8 mg kg^−1^ groups were not significantly different (0.061 and 0.7904, respectively; *p* > 0.05). Moreover, the recovery time of TOF ratio to 0.9 was shorter in the adamgammadex 10 mg kg^−1^ group than in the sugammadex 4 mg kg^−1^ group. Although the dose was increased from 6 to 8 and 10 mg kg^−1^, the recovery time of the TOF ratio to 0.9 was not significantly shortened (0.1017 and 0.2068, respectively; *p* > 0.05; Figure 3). The recovery time of the TOF ratio to 0.8 and 0.7 was similar to that of the TOF ratio to 0.9 and was dose-dependent (Figures 4, 5).

**Table 3:** The time of TOFr reaching 0.9 after the administration of muscle relaxants (mins).

	**Adamgammadex**					**Sugammadex**
	**2 mg/kg**	**4 mg/kg**	**6 mg/kg**	**8 mg/kg**	**10 mg/kg**	**4 mg/kg**
	**(*n* = 3)**	**(*n* = 4)**	**(*n* = 4)**	**(*n* = 5)**	**(*n* = 6)**	**(*n* = 5)**
Mean ± SD	13.31 ± 9.39^*^	8.11 ± 6.15^*^	3.67 ± 0.77	2.88 ± 0.49	2.35 ± 0.91	2.79 ± 0.44
Median	10.73	5.99	3.48	3.23	1.98	2.73
Mini, maximum	5.47,23.72	3.48,16.97	2.98,4.72	2.23,3.23	1.48,3.48	2.23,3.23

*TOFr ≥0.9 for three consecutive times is considered to be TOFr ≥0.9*.

*The first time when TOFr ≥0.9 was recorded as the time of TOFr reaching 0.9*.

* *Significantly prolonged compared with the active control group*.

After 3 min of adamgammadex 8 and 10 mg kg^−1^ and sugammadex 4 mg kg^−1^ administration, the average value of the TOF ratio was over 90% (TOF ratio ≥0.9) (Table 4). The mean value of the TOF ratio in the sugammadex 4 mg kg^−1^ group was similar with that in the adamgammadex 8 mg kg^−1^ dose group but was smaller than that in the adamgammadex 10 mg kg^−1^ group. This result meant that the recovery rate was faster with adamgammadex 10 mg kg^−1^ than with sugammadex 4 mg kg^−1^. In addition, the average values of the TOF ratio after 5 min of adamgammadex 6, 8, and 10 mg kg^−1^ and sugammadex 4 mg kg^−1^ administration were all above 90% (TOF ratio ≥0.9). At 10 min after administration, all dose groups, except the adamgammadex 2 mg kg^−1^ group, had a mean TOF ratio of ≥0.9. Remarkably, all the mean TOF ratio values were 100% (TOF ratio ≥1.0) in the adamgammadex 6, 8, and 10 mg kg^−1^ and sugammadex 4 mg kg^−1^ dose groups.

**Table 4:** The recovery value of TOFr 3, 5, and 10 min after muscle relaxant antagonist administration.

	**Adamgammadex**					**Sugammadex**
	**2 mg/kg**	**4 mg/kg**	**6 mg/kg**	**8 mg/kg**	**10 mg/kg**	**4 mg/kg**
	**(*n* = 3)**	**(*n* = 4)**	**(*n* = 4)**	**(*n* = 5)**	**(*n* = 6)**	**(*n* = 5)**
3 min after administration						
*n*	2	4	4	5	6	5
TOFr	51.5 ± 6.36	45.0 ± 27.48	76.3 ± 18.96	91.8 ± 5.72	99.2 ± 12.42	93.2 ± 5.02
5 min after administration						
*n*	2	4	4	5	6	5
TOFr	78.5 ± 12.02	83.3 ± 20.43	98.3 ± 4.57	105.2 ± 11.78	102.3 ± 8.29	91.8 ± 11.17
10 min after administration						
*n*	3	4	4	5	6	5
TOFr	89.7 ± 10.02	94.5 ± 10.63	103.3 ± 5.19	101.4 ± 3.13	103.5 ± 7.71	95.0 ± 4.47

The symptoms of recurarization (i.e., relapse of NMB caused by insufficient reversal effect) manifested in one subject in the sugammadex 4 mg kg^−1^ group. There was no recurarization in any of the adamgammadex cases.

### Safety

There were no SAEs, and no subjects withdrew from the trial or died because of AEs. Among those who received adamgammadex, 25 subjects experienced a total of 58 AEs. In the active control group, six subjects experienced a total of 12 AEs (Table 5).

**Table 5:** Summary of adverse events (AEs) - Safety Analysis Set.

	**Adamgammadex**												**Sugammadex**	
	**2 mg/kg (*****n*** **=** **6)**		**4 mg/kg (*****n*** **=** **5)**		**6 mg/kg (*****n*** **=** **6)**		**8 mg/kg (*****n*** **=** **6)**		**10 mg/kg (*****n*** **=** **6)**		**Summation (*****N*** **=** **29)**		**4 mg/kg (*****n*** **=** **6)**	
	**Events**	**Subjects with at least one adverse event (%)**	**Events**	**Subjects with at least one adverse event (%)**	**Events**	**Subjects with at least one adverse event (%)**	**Events**	**Subjects with at least one adverse event (%)**	**Events**	**Subjects with at least one adverse event (%)**	**Events**	**Subjects with at least one adverse event (%)**	**Events**	**Subjects with at least one adverse event (%)**
Total adverse events	8	4 (66.7%)	10	5 (100.0%)	9	5 (83.3%)	12	5 (83.3%)	19	6 (100.0%)	58	25 (86.2%)	12	6 (100.0%)
Various laboratory examinations	8	4 (66.7%)	9	5 (100.0%)	8	5 (83.3%)	12	5 (83.3%)	16	5 (83.3%)	53	24 (82.8%)	10	6 (100.0%)
Urinary tract infection	0	0	0	0	1	1 (16.7%)	0	0	2	2 (33.3%)	3	3 (10.3%)	0	0
Abdominal discomfort	0	0	1	1 (20.0%)	0	0	0	0	0	0	1	1 (3.4%)	0	0
Sinus bradycardia	0	0	0	0	0	0	0	0	1	1 (16.7%)	1	1 (3.4%)	0	0
Recurrence of neuromuscular block	0	0	0	0	0	0	0	0	0	0	0	0	1	1 (16.7%)
Shiver	0	0	0	0	0	0	0	0	0	0	0	0	1	1 (16.7%)

*According to the frequency, the adverse events related to various examinations were as follows: elevated serum creatine phosphokinase, urine ketone body positive, elevated blood ketone body, urine red blood cells positive, etc*.

In most subjects in the adamgammadex dose groups and the sugammadex 4 mg kg^−1^ group, the laboratory examinations showed abnormal but clinical insignificant results; those with clinical significance were recorded as AEs. In the adamgammadex groups, there were changes from the baseline in the hematology, biochemistry, and urinalysis results; these included elevated serum creatine phosphokinase, presence of urinary ketone body and glucose, positive urine red blood cells, elevated conjugated bilirubin, and urinary tract infection. In the active control group, the most common AEs were elevated blood ketone body, elevated blood glucose, elevated serum creatine phosphokinase, presence of urinary ketone body, positive urine red blood cells, elevated conjugated bilirubin, elevated blood bilirubin, incomplete reversal of NMB, and shivering.

Only one subject experienced AE that was considered possibly related with adamgammadex. After 2–5 min of administration of 10 mg kg^−1^ adamgammadex, that patient who had no preexisting cardiac illnesses developed grade 2 sinus bradycardia, which resolved shortly after atropine treatment. Another subject in the sugammadex 4 mg kg^−1^ group experienced grade 1 recurrent NMB (recurarization), which was reversed and recovered after close muscle relaxation monitoring and continuous mechanical ventilation, with no further intervention.

Overall, adamgammadex had a low incidence rate of AEs and good tolerance, with no recurarization and no AEs of allergy, vomiting, nausea, hypotension, and headache, which are common in similar drugs. The AE of sinus bradycardia with adamgammadex 10 mg kg^−1^ needs to be evaluated in a follow-up study to discover its relationship with the drug.

## Discussion

The main finding of this study was that after a single injection of adamgammadex at 2, 4, 6, 8, and 10 mg kg^−1^ doses at a post-tetanic count of 1–2, the time of recovery to a TOF ratio of 0.9 was dose-dependent, and there was a substantial decrease in the recovery time with increasing doses of adamgammadex. The current safety data showed that adamgammadex had a low incidence rate of AEs and good tolerance, in comparison with the well-established use of 4 mg kg^−1^ sugammadex.

Residual NMB following surgery and anesthesia has been a significant safety issue in the post-anesthesia care unit for decades and remains unsolved (20). Traditionally, the routine use of AChE antagonists has been proven ineffective in reversing profound and deep levels of NMB, not to mention the potential AEs of bradycardia, salivation, bronchospasm, and vomiting, which may be triggered by activating muscarinic acetylcholine and nicotinic receptors in other tissues (6, 7).

Because of the limitations of AChEs as antagonists of NMB, an alternative method of pharmacologic antagonism is clearly needed. In 2006, the SRBA sugammadex was first introduced by several researchers (10, 11, 21). Sugammadex is a modified γ-cyclodextrin that forms a complex with the aminosteriod NMBA molecules in a 1:1 ratio, instead of indirectly antagonizing NMBAs at the motor end-plate, thereby the binding of the NMBA to the receptor is prevented. The encapsulated NMBA complex has a very low disassociation rate and is excreted precipitously by the kidney. However, sugammadex has a relatively low molecular binding specificity because of the absence of chirality. Moreover, although sugammadex was commercially available in 2008, the United States FDA approved its clinical use only in late 2015. The major factor for the delay was the potential side effects of allergic reactions and post-operative bleeding (15, 16). The introduction of chiral carbons to cyclodextrin shows promise in improving the molecular binding specificity, which can further reduce the potential side effects.

Adamgammadex was developed exclusively by Hangzhou Adamerck Pharmlabs, Inc. A chiral acetyl amino group is introduced to the α carbon next to the carboxylic acid of each side chain of γ-cyclodextrin; this resulted in the addition of at least 20% of chiral carbon atoms and tremendously improved specificity (22). As a newly developed derivative of cyclodextrin, adamgammadex shares the same nucleus with sugammadex in the structure, which comprises a lipophilic core and a hydrophilic periphery; this provides a hydrophobic cavity for the encapsulation of free rocuronium or vecuronium molecules (23, 24). On isothermal titration calorimetry, adamgammadex was proven to reverse rocuronium-induced NMB at an equivalent molar ratio (17). Moreover, in preclinical pharmacological and toxicological studies on zebrafish and beagle dog models, adamgammadex achieved similar efficacy with sugammadex in reversing rocuronium-induced NMB. Moreover, the potential side effects, such as sensitization, bleeding, and heart rate changes, had lower incidence rates with adamgammadex than with sugammadex (18). Therefore, the results of preclinical studies demonstrated that adamgammadex had the potential to be an effective and safe option for clinical practice.

In a phase I clinical trial by Jiang et al. (19), no SAEs, baseline changes in vital signs, or clinically meaningful ECG abnormalities were observed after a single injection of adamgammadex to healthy volunteers. All subjects who experienced AEs recovered without any treatment or intervention. The incidence of AEs in the adamgammadex dose group was similar to that in the placebo control group, with no AEs judged as specific to adamgammadex. Compared with sugammadex, adamgammadex had a lower half-life (1.8 h) and higher urinary excretion ratio (83%), which meant lower potential risks of drug accumulation (21, 25, 26).

In this study, the subjects received a rapid intravenous injection of different doses of adamgammadex (2, 4, 6, 8, and 10 mg kg^−1^) when the TOF-Watch SX reading indicated deep NMB. Consistent with the results from animal experiments and the phase I clinical trial, the results of this study showed that adamgammadex can remarkably reverse the deep NMB induced by rocuronium. Compared with sugammadex 4 mg kg^−1^, which is the recommended dose for rocuronium-induced deep NMB (27), adamgammadex 2 and 4 mg kg^−1^ had longer recovery time of TOF ratio to 0.9, which varied considerably among individuals (25, 26). Some subjects who were given adamgammadex 2 mg kg^−1^ even failed to completely recover from NMB (TOF ratio ≥0.9) within 30 min (25, 26). Using a 1:1 binding model, adamgammadex was shown to selectively form an inactive and tight complex with rocuronium, suggesting that administration of adamgammadex at a dose below 4 mg kg^−1^ might be insufficient to encapsulate all of the free rocuronium molecules and adequately antagonize deep NMB (28). The efficacy of adamgammadex at 6, 8, or 10 mg kg^−1^ was similar to that of sugammadex at 4 mg kg^−1^. Based on our results on recovery time with the increasing doses of adamgammadex (Figure 3), adamgammadex in the dose range of 6–10 mg kg^−1^ may approach the plateau of the dose–response curve. Therefore, we hypothesized that 6–10 mg kg^−1^ might be the recommended dose range of adamgammadex for further efficacy exploration and that administration of adamgammadex at 8 and 10 mg kg^−1^ may have relatively good effects on antagonizing rocuronium-induced deep NMB.

Anaphylaxis and anticoagulant effects are the two common AEs of sugammadex use (29). Min et al. (30) and de Kam et al. (31) recently reported striking incidences of sugammadex-induced hypersensitivity in healthy volunteers, accounting for 6.6 and 0.7%, respectively, with 4 mg kg^−1^ and 9.5 and 4.7%, respectively, with 16 mg kg^−1^. Moreover, in patients who received sugammadex, activated partial thromboplastin time and prothrombin time were found to increase by 5.5 and 3.0%, respectively (15). Consistent with the results of the phase I safety assessment, the safety data from this phase IIa study indicated that adamgammadex had few side effects and was well-tolerated. Side effects, such as reoccurrence of NMB, incision pain, vomiting, nausea, hypotension, and headache, were not observed with adamgammadex (32–34). However, it is worth noting that one case of sinus bradycardia was observed with adamgammadex 10 mg kg^−1^; further studies are required to better characterize the safety profile of adamgammadex.

The small sample size was inevitably a limitation of the present study. Moreover, eight subjects had at least one major protocol violation and were excluded from the efficacy evaluation, leading to a smaller sample size. Nevertheless, in this pilot dose-finding study, the investigators did not find diverse TOF ratio values among the dose groups. The dose–response curve was clear, especially in the dose range of 6–10 mg kg^−1^, which was believed to be a reliable target for further efficacy exploration. Because the study was designed to establish a suitable dose for use in phase IIb and III studies, the investigators did not include the adamgammadex 8 and 10 mg kg^−1^ groups at the beginning of the study, and the allocation of these two higher dose groups (8 and 10 mg kg^−1^) did not precisely follow randomized allocation. Another limitation was the gender imbalance of the subjects in group 2 (adamgammadex 4 mg kg^−1^) and group 6 (sugammadex 4 mg kg^−1^); this will be avoided in our ongoing IIb study.

In conclusion, this study demonstrated that adamgammadex quickly, predictably, and safely reversed deep NMB of I–II PTCs in subjects categorized as ASA class I–II. Adamgammadex doses of ≤ 4 mg kg^−1^ were insufficient in antagonizing deep NMB, whereas doses of 6–10 mg kg^−1^ might be the recommended range for further efficacy exploration.

## Data Availability Statement

The original contributions presented in the study are included in the article/supplementary material, further inquiries can be directed to the corresponding author.

## Ethics Statement

The studies involving human participants were reviewed and approved by Ethics Committee of Renji Hospital. The patients/participants provided their written informed consent to participate in this study.

## Author Contributions

YQ, JQ, WY, and DS: conception and design. XH, ZY, and YZ: acquisition of data. XH, ZY, YZ, and SC: analysis and interpretation of data. SC and YZ: drafting of the article. All authors read and approved the final version of the manuscript.

## Conflict of Interest

YQ and JQ were employed by the company Hangzhou Adamerck Pharmlabs Inc. The remaining authors declare that the research was conducted in the absence of any commercial or financial relationships that could be construed as a potential conflict of interest.

## Publisher's Note

All claims expressed in this article are solely those of the authors and do not necessarily represent those of their affiliated organizations, or those of the publisher, the editors and the reviewers. Any product that may be evaluated in this article, or claim that may be made by its manufacturer, is not guaranteed or endorsed by the publisher.

## Correction note

This article has been corrected with minor changes. These changes do not impact the scientific content of the article.
